# Conceptualizing the Mechanisms of Social Determinants of Health: A Heuristic Framework to Inform Future Directions for Mitigation

**DOI:** 10.1111/1468-0009.12642

**Published:** 2023-04-16

**Authors:** MARCO THIMM‐KAISER, ADAM BENZEKRI, VINCENT GUILAMO‐RAMOS

**Affiliations:** ^1^ Center for Latino Adolescent and Family Health Duke University; ^2^ School of Nursing, Duke University; ^3^ School of Medicine, Duke University; ^4^ Presidential Advisory Council on HIV/AIDS, US Department of Health and Human Services

**Keywords:** social determinants of health, health inequities, framework, health policy, health programs, health system transformation

## Abstract

**Policy Points**

- A large body of scientific work examines the mechanisms through which social determinants of health (SDOH) shape health inequities. However, the nuances described in the literature are infrequently reflected in the applied frameworks that inform health policy and programming.
- We synthesize extant SDOH research into a heuristic framework that provides policymakers, practitioners, and researchers with a customizable template for conceptualizing and operationalizing key mechanisms that represent intervention opportunities for mitigating the impact of harmful SDOH.
- In light of scarce existing SDOH mitigation strategies, the framework addresses an important research‐to‐practice translation gap and missed opportunity for advancing health equity.

**Context:**

The reduction of health inequities is a broad and interdisciplinary endeavor with implications for policy, research, and practice. Health inequities are most often understood as associated with the social determinants of health (SDOH). However, policy and programmatic frameworks for mitigation often rely on broad SDOH domains, without sufficient attention to the operating mechanisms, and effective SDOH mitigation strategies remain scarce. To expand the cadre of effective SDOH mitigation strategies, a practical, heuristic framework for policymakers, practitioners, and researchers is needed that serves as a roadmap for conceptualizing and targeting the key mechanisms of SDOH influence.

**Methods:**

We conduct a critical review of the extant conceptual and empirical SDOH literature to identify unifying principles of SDOH mechanisms and to synthesize an integrated framework for conceptualizing such mechanisms.

**Findings:**

We highlight eight unifying principles of SDOH mechanisms that emerge from landmark SDOH research. Building on these principles, we introduce and apply a conceptual model that synthesizes key SDOH mechanisms into one organizing, heuristic framework that provides policymakers, practitioners, and researchers with a customizable template for conceptualizing and operationalizing the key SDOH mechanisms that represent intervention opportunities to maximize potential impact for mitigating a given health inequity.

**Conclusions:**

Our synthesis of the extant SDOH research into a heuristic framework addresses a scarcity of peer‐reviewed organizing frameworks of SDOH mechanisms designed to inform practice. The framework represents a practical tool to facilitate the translation of scholarly SDOH work into evidence‐based and targeted policy and programming. Such tools designed to close the research‐to‐practice translation gap for effective SDOH mitigation are sorely needed, given that health inequities in the United States and in many other parts of the world have widened over the past two decades.

The reduction of population‐level health inequities has become a broad and interdisciplinary endeavor with implications for policy, research, and practice in health care,^1^ public health,^2^ biomedicine,^3^ criminal justice,^4^ education,^5^ business,^6^ and beyond (see the definition of “health inequity” and the glossary of other key terms in Box 1). Increasingly, the scientific literature understands and explains chronic and contemporary health inequities, disparities in health that are assumed to be systematic, unfair, and avoidable (although the terminology for the concept has been inconsistently defined and used, we use “health inequities”^7^), as being associated with the social determinants of health (SDOH).^5^, ^8^ As such, formal frameworks that operationalize SDOH as the underlying drivers of health inequities are important tools for decision makers to inform health‐related planning and programming. For example, the *Healthy People 2030* plan in the United States and the *Addressing Health Inequalities in the European Union* report draw on frameworks of SDOH to guide programmatic efforts aimed at reducing health inequities.^9^, ^10^

**Box1. Glossary of Key Terms**

**Health**: Defined by the World Health Organization as “a state of complete physical, mental, and social well‐being and not merely the absence of disease or infirmity.”^93^

**Health disparity**: A difference in health outcomes among segments of the population, as defined by social, demographic, environmental, or geographic attributes.^142^

**Health inequity**: A health disparity that is assumed to be produced through systematic, unfair, and avoidable causal mechanisms.^7^

**Social determinants of health**: Defined by the World Health Organization as “the conditions in which people are born, grow, work, live, and age and the wider set of forces and systems shaping the conditions of daily life [and health outcomes].”^143^

**Social determinants of health capital**: Resources and opportunities that are socially allotted to persons or groups and that affect health outcomes (e.g., income, educational attainment, quality of housing, accessibility and quality of available health care).

**Social determinants of health processes**: Social factors shaping the interactions among persons, groups, institutions, or systems that affect health outcomes (e.g., policies, norms, racism, sexism, classism, xenophobia, homophobia).

**Exposure**: Subjection to a health risk or protective factor (exposure is environmental or behavioral).

**Susceptibility**: The likelihood of morbidity/mortality given the exposure to a health risk factor (susceptibility is biological).

**Resilience**: Collective action to reduce the harmful impact of structural adversity on individuals, institutions, and communities/populations' ability to thrive (resilience does not solely rely on the strengths of individuals and communities and requires societal investments in the multilevel systems that are responsible for advancing health equity).

It is important to note that these prominent policy and programmatic frameworks of SDOH impact frequently rely on broad domains of SDOH, such as income, educational attainment, quality of housing, health care access, and racial/ethnic discrimination, which are conceptualized as predicting a range of health inequity outcomes.^9^, ^10^, ^11^, ^12^, ^13^ A large body of conceptual and empirical literature highlights the relevance of these domains of SDOH with respect to shaping health outcomes and inequities along a gradient of stratification based on social position.^14^, ^15^, ^16^, ^17^, ^18^, ^19^ For example, socioeconomic status is widely considered predictive of a variety of positive and negative health outcomes.^14^, ^19^ Similarly, the positive correlation between level of education and overall health is well documented.^15^ However, applied policy or programmatic frameworks that rely primarily on broad domains of SDOH (hereinafter referred to as “domain‐focused” frameworks) seldom specify the operating mechanisms of SDOH in sufficient detail to guide targeted intervention, and rigorously evaluated and effective programmatic strategies for mitigating the mechanisms of SDOH impact remain scarce.^20^, ^21^

This is despite a large body of conceptual and empirical work on SDOH mechanisms in the extant literature. Several influential SDOH theories have emerged, including the fundamental causes theory, the risk environment theory, the syndemic theory, the ecosocial theory, and life‐course and biological approaches for understanding the mechanisms of SDOH, among others.^22^, ^23^, ^24^, ^25^, ^26^, ^27^, ^28^, ^29^, ^30^ In addition, numerous frameworks have collectively advanced efforts to integrate the SDOH literature for use in research and applied work, including the widely cited SDOH framework developed by the World Health Organization Commission on Social Determinants of Health, the National Institute on Minority Health and Health Disparities (NIMHD) research framework, frameworks of health and health equity drivers reviewed by Givens and colleagues, as well as others.^31^, ^32^, ^33^, ^34^, ^35^, ^36^ Still, to date, the nuances of SDOH mechanisms described in the literature are too often not reflected in the broad, SDOH domain–focused frameworks that continue to inform health policy, public health, and clinical practice, representing a research‐to‐practice translation gap and missed opportunity for more effective SDOH mitigation.

## Moving Toward a Strength‐Based Paradigm for Addressing Health Inequities

The increasing prioritization of SDOH in a broad range of disciplines has motivated a large and growing number of researchers to focus on the identification of population‐level health inequities^14^, ^37^, ^38^ and the description of affected groups and communities in terms of sociodemographic factors, such as race/ethnicity, socioeconomic status, level of education, biological sex, gender identity, sexual orientation, etc., often characterizing them as “vulnerable.”^14^, ^38^, ^39^, ^40^ As a consequence, vulnerability to negative health outcomes has frequently served as a surrogate explanatory construct for understanding the impact of SDOH on health inequities. However, characterizations of vulnerability often fail to elucidate the operating mechanisms of SDOH and contribute little to informing applied intervention.^39^, ^40^

Vulnerability‐focused conceptualizations of SDOH impact reflect a deficiency‐focused perspective on health inequities; that is, the approaches for describing, explaining, and addressing health inequities tend to be guided by the question *What makes communities do poorly?* ^39^ Within this paradigm, researchers seek to identify and practitioners seek to address structural disadvantages (i.e., the harmful SDOH) and mediating risk factors (i.e., vulnerability factors) that are predictive of negative health outcomes. An alternative but less common question for guiding health inequity research and programming is *What makes communities do well despite significant structural challenges?* ^39^ This change of perspective represents a worthwhile departure from deficiency‐focused paradigms of thought, as it emphasizes the identification of protective levers to mitigate health inequities within a context of chronic and persistent social injustice.

At the same time, it is important to recognize that strength‐based interventions focused primarily on offsetting the negative effects of harmful SDOH should not be viewed as a substitute for broader efforts to eliminate the underlying drivers of health inequities. Effective approaches to reducing the negative impact of harmful SDOH will require a simultaneous focus on prioritizing the elimination of the underlying drivers of chronic health inequities while also identifying programmatic opportunities in policy, public health, and health care practice that can be amplified to reduce the health impact of underlying drivers while they persist, including by investing in strategies for health and wellbeing that are indigenous to communities experiencing harmful SDOH. Within this paradigm, tools are needed to support translation of scholarly SDOH work into effective health policy and programming.

To this end, we propose and apply a practical, heuristic framework that provides policymakers, practitioners, and researchers with a customizable template for conceptualizing and operationalizing the key SDOH mechanisms that represent leverage points—intervention opportunities to maximize potential impact in mitigating a given health inequity. The framework serves as a tool to facilitate the translation of scholarly SDOH work into effective health policy, programming, and clinical care, thereby addressing a scarcity of peer‐reviewed organizing frameworks of SDOH mechanisms that are explicitly designed to inform practice.^36^

## Unifying Principles for the Mechanisms of SDOH

As a starting point for the synthesis of an organizing framework of SDOH mechanisms that is grounded in the extant literature, we conducted a critical review of seminal SDOH research, following guidance laid out by Grant and Booth^41^ to identify principles that are reflected across influential SDOH theories and those that have utility for establishing linkages between frameworks. For conceptual synthesis, we drew on approaches for theory construction outlined by Jaccard and Jacoby.^42^ Specifically, the authors used a process‐oriented approach to analyze the identified literature during open‐ended discussions. Our process‐oriented synthesis of the literature sought to posit general rules by which dynamic systems of SDOH mechanisms tend to behave in shaping health inequities, as opposed to a variable‐oriented approach focused on delineating a more static set of specific causal factors.^42^ We highlight eight unifying principles for the mechanisms of SDOH that emerged from the critical review and conceptual synthesis. Alongside each principle, we discuss the core elements of an influential SDOH theory that serve to illustrate the central tenets of the respective principle but do not present a comprehensive discussion of each theory or of all relevant theories, which is beyond the scope of this paper.

### SDOH Are Underlying Causes of Health Inequities

Most SDOH frameworks and theories approach the explanation of health inequities by going beyond considering only proximal risk factors.^43^, ^44^ Instead, these frameworks consider a set of underlying social factors as most influential in shaping health inequities. Link and Phelan's fundamental causes theory represents an early and influential articulation of this principle.^19^, ^22^ The theory posits that health inequities arise as a consequence of social stratification based on wealth, power, and prestige, primarily along the lines of socioeconomic status.^19^, ^22^ In socioeconomic strata with a lower allotment of health‐related capital, these underlying social causes systematically increase the density of disease risk factors and reduce access to resources that represent disease protective factors, producing inequities across multiple health outcomes.^19^, ^22^ A large body of literature empirically supports this principle, showing that these mechanisms result in inequitable health outcomes in communities of low socioeconomic status over time.^19^, ^45^

The growing recognition of underlying “fundamental causes” as principal drivers of persistent health inequities in underserved communities has been an important contributor to the increasing research, programmatic, and policy focus on SDOH in recent years. The growing emphasis on SDOH as driving health inequities represents a departure from prior explanatory frameworks that primarily relied on individual agency for understanding negative health outcomes (e.g., decisions regarding health), underappreciating the absence of equal opportunity for achieving optimal health. Instead, SDOH emerge as structural drivers, or fundamental causes, of health inequities, with individuals, communities, or populations experiencing harmful SDOH being at increased risk of negative health outcomes, largely owing to scarce health‐related resources and opportunities.^19^, ^22^, ^45^, ^46^, ^47^, ^48^

### SDOH Shape Health Inequities Through Contextual Influences

The recognition of health risk factors that are exogenous to the individual, as reflected in the growing focus on the mitigation of SDOH through structural interventions,^49^ represents another key principle reflected in the SDOH literature. There is a long history of regarding health risks as a function of the individual, including in biomedical models of disease that prioritize proximal risk factors at the individual level (e.g., behavior, genetics) and some social epidemiological models that prioritize more distal, yet individual‐level, risk factors (e.g., income, educational attainment). However, the environmental production of risk has been increasingly adopted as an explanatory concept for health inequities and warrants consideration as an independent principle.^5^ The risk environment framework advanced by Rhodes and colleagues serves to effectively illustrate this principle.^23^, ^24^ The risk environment is conceptualized as the physical, social, economic, and political structures and influences that exist outside of the individual but which produce or reduce individual disease risk given that individuals are shaped by and interact with their environment in a multitude of ways.^23^ As underlying drivers of health inequities, SDOH operate in part by shaping these physical, social, economic, and political conditions across communities.

Importantly, environmental risk cannot always be considered independently of behavioral risk because the health risk associated with certain behaviors is often dependent on the environment where the behavior occurs (e.g., risk of HIV infection associated with unprotected sex in a context of high community viral load vs. low community viral load),^50^ and because environmental influences have been shown to shape behavior itself (e.g., paraphernalia laws shaping drug injection practices).^23^ Given that individuals from the same community are likely to experience similar environmental influences and risk environments, negative health outcomes tend to cluster to produce health inequities.

### SDOH Contextual Disadvantage Is Not Deterministic

Further, the extant SDOH research facilitates understanding of another particularly important principle of SDOH mechanisms; despite robust and consistent evidence of the clustering of negative health outcomes in certain socioeconomic strata or community contexts,^43^, ^46^, ^51^ the harmful influences of distal drivers of health inequities, such as low educational attainment, and of contextual disadvantages, such as low community‐level density of health services, do not immutably cause negative health outcomes. For example, individuals with the same educational background frequently have different health outcomes, and communities with the same density of health services frequently have different base rates of morbidity and mortality.

Several influential SDOH frameworks emphasize the importance of complex systems (i.e., nonlinear, multilevel, and dynamic influences and mechanisms) in shaping both individual health and the emergence of community‐level health inequities.^22^, ^23^, ^24^, ^25^, ^26^, ^27^, ^28^, ^29^, ^30^, ^31^ Although the impact of harmful SDOH will, on average, result in worse health outcomes, the mechanisms through which harmful SDOH influence operates and the degree to which the associated negative health impacts ultimately manifest can differ among individuals and communities because health outcomes are shaped by numerous mechanisms across several levels of influence simultaneously. For example, the risk environment framework outlines the interplay of influences at three levels that together shape health risk and outcomes—the micro (individual), meso (institutional), and macro (community/societal) levels.^24^ The nature of influences at each level can either protect from or increase the risk of disease, and multilevel influences interact to reinforce or weaken the effects of one another. Therefore, similar macro, meso, or micro contexts of health risk do not always produce identical health outcomes. For example, work examining social capital and social cohesion in relation to health has highlighted the important protective influence of relationships among actors both within and across levels, including individual networks (micro level), institutions (meso level), and community/societal groups (macro level),^31^ which is reflected in the growing recognition of meaningful community engagement as a key opportunity for improving health equity research and programming.^52^ Altogether, greater emphasis on understanding the factors that offset the negative impacts of harmful SDOH can inform optimally effective mitigation efforts.

### SDOH Shape Health Over the Life Course

The life‐course framework in SDOH research suggests that social, economic, psychological, environmental, and other influences accumulate over the life course to shape health behavior and mental and physical health.^29^, ^53^ As such, early‐life adverse or protective SDOH affect health outcomes later in life. Notably, a comprehensive body of research has examined the various aspects of SDOH influences over the course of life, including work on the fetal origins of disease,^54^ sensitive developmental periods,^55^ scaffolding theory,^56^ and the long‐term impacts of trauma and allostatic load.^57^ Two overarching mechanisms for life course–related SDOH impact stand out as generalizable.^29^ First, at any given life stage, SDOH shape exposure to risk or protective factors. However, the health consequences attributable to a given risk or protective factor may manifest later in life.^29^ For example, research conducted by the US Centers for Disease Control and Prevention suggests that nearly half (44%) of all cases of depression among adults may be attributable to adverse experiences during childhood.^58^ Second, the nature of SDOH influences that occur early in life predict the nature of SDOH influences later in life. For example, children who grow up in poverty are more likely to experience poverty in adulthood.^59^ The persistence of SDOH influences over the course of life promotes the accumulation of exposures and associated health consequences.^29^

### SDOH Operate Through Biological Embedding

The influence of SDOH on the distribution of various contextual health risk and protective factors is well documented and is largely accepted as a core mechanism of SDOH impact. In addition, previous research conceptually and empirically supports the role of SDOH in shaping biological processes that contribute to negative health outcomes (biological embedding).^26^, ^27^, ^28^, ^29^, ^30^, ^60^ Biological embedding is defined as the process by which SDOH initiate and sustain biological changes that are associated with an increased likelihood of negative short‐ and long‐term physical or mental health outcomes.^60^ Biological embedding that operates through epigenetic, neurodevelopmental, immune, endocrine, and microbiome changes has been described.^60^, ^61^, ^62^ Accelerated cardiometabolic aging, which is associated with residing in contexts of disadvantage, represents an example of biological embedding.^63^ In addition, adverse experiences during childhood have been shown to impact neurodevelopment, including brain structure (e.g., decreased hippocampal volume and gray matter) and function (e.g., amygdala functional connectivity),^64^, ^65^ representing another manifestation of biological embedding. Biological embedding can be understood as the continual, dynamic interactions of the social and physical environment with biological processes that occur in the body and brain over the life course.^66^, ^67^, ^68^, ^69^ As such, the life course–related impact of SDOH should be conceptualized not only as the accumulation of environmental health risk factors but also of biological changes that are associated with increased morbidity and mortality.

### SDOH Operate Intergenerationally

The impact of SDOH has been described not only as manifesting over the course of life but also as operating intergenerationally. The principle of intergenerational SDOH impact serves to conceptualize mechanisms that explain the clustering of negative health outcomes across generations beyond those that are attributable to genetic predisposition. The biosocial inheritance framework serves to effectively illustrate this principle.^67^, ^70^ Biosocial inheritance is defined as the transmission of social adversity and advantage across generations.^70^ It operates through both the socioenvironmental and biological mechanisms that have been described in previous sections. There are three types of intergenerational SDOH impacts that warrant consideration. Cross‐generational SDOH impact refers to social influences that lead to exposures at the parent level, with the resulting health consequences manifesting in the offspring (e.g., through exposure to environmental toxins or elevated levels of stress during pregnancy).^70^, ^71^, ^72^ Multigenerational SDOH impact refers to social influences that lead to exposures and corresponding health consequences that affect multiple generations simultaneously (e.g., through stressors such as poverty, food insecurity, or housing instability that affect both children and parents).^70^, ^73^ Transgenerational SDOH impact refers to social influences that lead to exposures for which the resulting health consequences are passed down to multiple generations (e.g., transmission of trauma through inheritance of epigenetic tags).^70^, ^74^

Undoubtedly, the family represents a particularly important health‐related context early in life, with parental influences having an outsized effect on child and adolescent health outcomes and trajectories. Although a context of harmful SDOH influences places a significant burden on the caregiving environment that can be associated with child and adolescent stressors,^75^, ^76^ parents and the family represent powerful protective buffers against the negative effects of environmental risk on the health and development of children and adolescents.^77^, ^78^, ^79^ For example, parental influences have been shown to offset associations of stressful life events, racial discrimination, and exposure to community violence with adolescent externalizing problems (e.g., unprotected sex, substance use) in families experiencing harmful SDOH.^80^ Thus, the family must be recognized as an important unit for both understanding and mitigating the impact of SDOH. This has implications for both research and practice paradigms, which, to date, have tended to focus on individuals rather than families.

### SDOH Shape Clustering and Synergies of Health Inequities

The principles of SDOH impact that identify fundamental causes and contextual influences in shaping health outcomes also help explain the clustering of health inequities in communities and populations that experience adverse SDOH. Building on these principles, the work advanced by Singer and Clair describes another key mechanism of SDOH impact—the synergistic interaction of coexisting health disparities.^25^ Syndemic theory focuses on the excess disease burden that is attributable to the synergistic interactions of two or more epidemics within the same community or population (i.e., the extent to which the combined morbidity attributable to multiple cooccurring epidemics is greater than the summed morbidity had each epidemic occurred separately).^25^ There are both social and biological mechanisms that can produce syndemic dynamics.^25^ For instance, an increased risk of HIV acquisition among people who use substances and engage in disinhibited sexual behavior is an example of sociocontextual synergism.^81^ Inflammation of the genital tissue caused by a sexually transmitted infection, which in turn facilitates the transmission or acquisition of HIV, is an example of biological synergism.^82^ Given that the likelihood of epidemiological synergies increases with a greater number of cooccurring epidemics, the impact of syndemics is the greatest in communities and populations in which many health disparities tend to cluster, namely those impacted by adverse SDOH. Amplifying clustering mechanisms of SDOH impact across multiple levels and across time also warrant mention. First, harmful and protective SDOH influences tend to cluster within and across individuals, families, schools, neighborhoods, communities, etc., with interactions across levels amplifying the impact of harmful SDOH.^83^ Second, social stratification in morbidity and disability contributes to further entrenching the existing social hierarchies in terms of wealth, power, and status,^83^, ^84^, ^85^ thereby amplifying SDOH impact and health inequities over time.

### Unjust Social Processes Shape SDOH Mechanisms to Produce Health Inequities

Last, seminal work on SDOH has examined the mechanisms that produce health inequities from the perspective of social processes. Krieger's ecosocial framework, which conceptualizes health inequities as biological expressions of unjust social processes, has been particularly influential in establishing this principle.^26^, ^27^, ^28^ Structural racism, the negative health impact of which is supported by a growing body of evidence,^86^ serves to illustrate the central tenets of the principle. Structural racism as an exploitative and oppressive social process shapes, and is shaped by, long‐standing racial/ethnic social inequality in various domains,^87^, ^88^ including those that in turn impact health (e.g., income, educational attainment, quality of housing, health care access).^14^ There is also evidence of a direct impact of racism on health, for example, through chronic stress and the associated inflammatory processes^89^ and through interactions between racism and other SDOH influences (e.g., structural racism not only reduces access to health care but also impacts the quality of care when care is accessed).^90^, ^91^ Therefore, social processes, such as structural racism, classism, sexism, xenophobia, and homophobia, including as expressed in policies and regulations, fundamentally shape the mechanisms through which SDOH affect health inequities,^87^, ^88^, ^91^ as well as the ways in which SDOH mechanisms are conceptualized, examined, and targeted for mitigation.^92^

**Figure 1:**
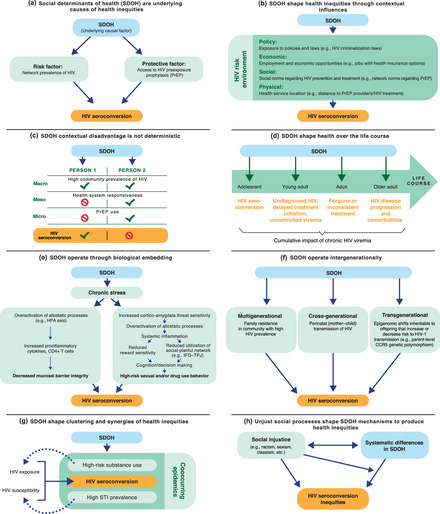
Eight Key Principles of Social Determinants of Health (SDOH) Mechanisms Shaping HIV Inequities. [Colour figure can be viewed at wileyonlinelibrary.com] Case examples for the application of the principles of SDOH mechanisms to HIV inequities. (**A**) SDOH act as underlying drivers of HIV transmission by shaping the distribution of HIV risk and protective factors.^22^, ^44^ (**B**) SDOH influence manifests in the policy, economic, social, and physical dimensions of the HIV risk environment.^23^, ^24^ (**C**) Adverse SDOH influences at the macro level (macro) are not deterministic in shaping HIV outcomes but interact with meso level (meso) and micro level (micro) influences.^24^ (**D**) SDOH influence in early life and over the life course shapes HIV treatment outcomes and disease progression.^29^, ^125^, ^126^ (**E**) The impact of SDOH on HIV outcomes includes biological mechanisms of influence,^60^, ^67^, ^68^, ^69^ including inflammatory^127^, ^128^, ^129^, ^130^, ^131^, ^132^, ^133^ and neural^69^, ^131^, ^132^, ^133^ mechanisms. (**F**) SDOH shape HIV outcomes for several generations through three types of intergenerational influence.^67^, ^69^, ^134^, ^135^, ^136^, ^137^ (**G**) SDOH shape the clustering of multiple cooccurring epidemics, which amplifies exposures and susceptibilities associated with increased HIV transmission.^25^, ^138^, ^139^ (**H**) Social injustice shapes HIV inequities directly and in interaction with systematic differences in other SDOH.^26^, ^27^, ^28^, ^47^, ^139^ ^.^ Abbreviations: CCR5, C‐C chemokine receptor type 5; CD4, cluster of differentiation 4; HPA, hypothalamic‐pituitary‐adrenal; IFG, inferior frontal gyrus; PrEP, HIV preexposure prophylaxis; STI, sexually transmitted infection; TPJ, temporo‐parietal junction.

Considered together, the eight principles identified here draw on influential SDOH research to highlight the key mechanisms through which the influence of SDOH on long‐standing and current health inequities can be conceptualized. In Figure 1, we illustrate each principle using applied examples of the mechanisms of SDOH shaping HIV inequities. In addition, the principles point to future directions for advancing systems transformation in health policy, public health, and clinical practice toward greater alignment with stated health equity goals.^1^, ^2^, ^3^, ^4^, ^5^, ^6^, ^9^, ^10^, ^11^, ^12^, ^13^, ^31^, ^93^ Table 1 highlights priorities for health equity–focused systems transformation that emerge from the eight principles of SDOH mechanisms, each contrasted against the current status quo.

**Table 1:** Priorities for Health Equity–Focused Systems Transformation (versus Status Quo) that Emerge from the Eight Unifying Principles of SDOH Mechanisms

**Principle of SDOH Mechanisms**	**Priority for Health Equity–Focused Systems Transformation (vs. Status Quo)**
SDOH are underlying causes of health inequities	Meaningful community engagement in data generation and interpretation for understanding and mitigating underlying health inequity drivers and multilevel resilience factors (vs. an exclusively data‐driven focus on identifying community risk factors)
SDOH shape health inequities through contextual influences	Development, evaluation, and scale up of multilevel interventions that address the mechanisms of SDOH at the structural, psychosocial, and clinical/biomedical levels (vs. primarily biomedical approaches combined with screening for individual social needs [e.g., housing, food, transportation] and subsequent referrals to psychosocial services)
SDOH contextual disadvantage is not deterministic	Adoption of individualized/differentiated, decentralized, and community‐based service delivery models (vs. centralized one‐size‐fits‐all models)
SDOH shape health over the life course	Proactive intervention focused on prevention and health promotion as well as restorative care to maintain and improve physical, mental, and psychosocial functioning and quality of life (vs. focus on treatment within a system built for sick care)
SDOH operate through biological embedding	Greater prioritization of harmful SDOH mechanisms and mitigation of their biological impact in clinical education and practice, including investment in biomarkers for early detection of and intervention on emerging disease trajectories (vs. focus on diagnosis after disease onset and subsequent disease management)
SDOH operate intergenerationally	Prioritization of family‐based approaches to restorative health care, prevention, and health promotion (vs. primarily individual‐focused intervention approaches)
SDOH shape clustering and synergies of health inequities	Greater integration of comprehensive, interdisciplinary, team‐based health services delivered within a value‐based framework and at the top of providers’ licenses (vs. fragmented and siloed fee‐for‐service systems with practice restrictions for nonphysician providers)
Unjust social processes shape SDOH mechanisms to produce health inequities	Departure from vulnerability‐ and deficiency‐focused paradigms for understanding health inequities toward multilevel resilience‐focused paradigms for reducing health inequities (i.e., “What makes communities do well despite significant structural challenges?” vs. sole focus on “What makes communities do poorly?”)

Abbreviations: SDOH, social determinants of health.

## An Organizing Framework of SDOH Mechanisms

**Figure 2:**
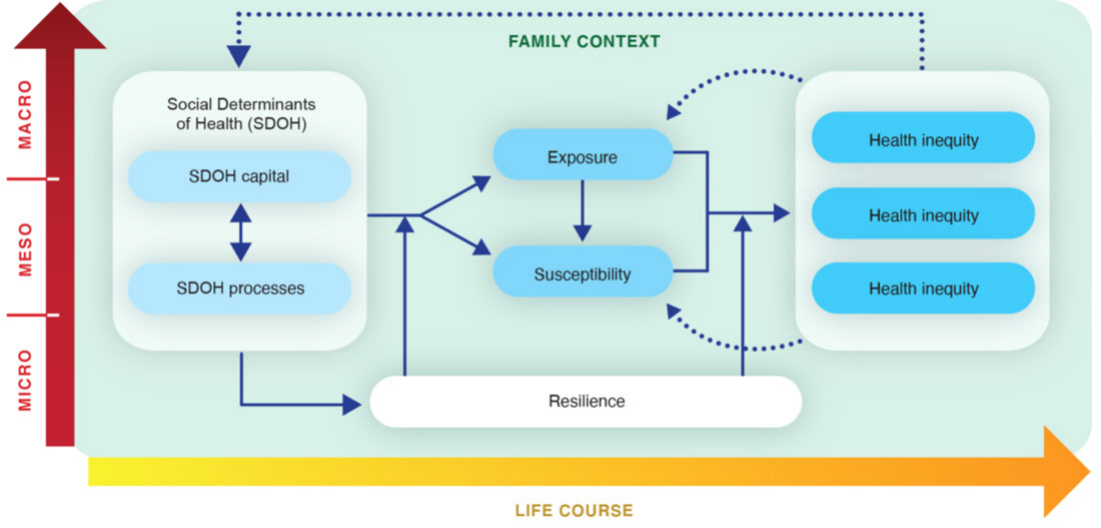
The Center for Latino Adolescent and Family Health Framework of SDOH Mechanisms. [Colour figure can be viewed at wileyonlinelibrary.com] Abbreviations: macro, macro level; meso, meso level; micro, micro level; SDOH, social determinants of health.

Building on the eight unifying principles outlined above, we propose a conceptual model that synthesizes the eight key SDOH mechanisms into one organizing framework (Figure 2), which is designed to serve as a roadmap for policymakers and applied researchers in conceptualizing leverage points for mitigating a given health inequity. The goal of our synthesis was to (1) distill clearly defined concepts that hold meaning for the future users of the framework (see the glossary of key terms in Box 1) and (2) put them into relationships with each other so as to capture the dynamic processes of SDOH mechanisms that are reflected in the eight principles.^42^ The resulting conceptual model incorporates SDOH as underlying causes that shape health inequities through nonlinear, mediated, and moderated relationships (Principle 1). More specifically, the model distinguishes between SDOH capital, the resources and opportunities that affect health outcomes and are socially allotted to individuals and groups (e.g., educational attainment, quality of housing, income), and SDOH processes, the often unjust social processes shaping the interactions and relationships among persons, groups, institutions, or systems that affect health outcomes (e.g., racism, policies, norms) (Principle 8). The influence of SDOH is mediated by exposure, which is conceptualized as context‐specific environmental and behavioral risk (Principle 2), and biologically embedded susceptibility (Principle 5). The depicted SDOH mechanisms are not deterministic given that the model incorporates the moderating influence of multilevel resilience factors (Principle 3). At the outcome level depicted at the right end of the figure, the model accounts for the clustering of multiple cooccurring health inequities and corresponding synergisms that reinforce the impact of SDOH (dotted arrows) (Principle 7). In addition, the model assumes that the influence of the outlined SDOH mechanisms accumulates over the course of life (Principle 4) and recognizes the family as a primary context for understanding and mitigating the intergenerational impact of SDOH (Principle 6). Last, the model considers the depicted constructs and relationships as operating across multiple levels and encourages operationalizing them as such.

The proposed framework of SDOH mechanisms offers a rich, yet generalizable, template for conceptual, empirical, and applied work designed to develop and implement comprehensive and multilevel interventions, programs, and policies to prevent and mitigate the harmful impact of SDOH. Specifically, our framework serves as a heuristic for conceptualizing four key elements of SDOH mechanisms: underlying causal factors, mediating factors, moderating factors, and health inequity outcomes.

### Underlying Factors

Our proposed framework conceptualizes SDOH—the distal causal factors that shape health inequities—as two distinct classes of social influence. Specifically, we distinguish between *SDOH capital* and *SDOH processes*. We conceptualize SDOH capital as quantifiable resources and opportunities that affect health outcomes, for which allotment to individuals or groups is primarily determined by social factors. For example, educational attainment can be measured at the level of the individual or the community, is important for shaping health outcomes, and is largely distributed along a social gradient. In contrast, we conceptualize SDOH processes as social factors shaping the interactions among persons, groups, institutions, or systems that impact health. The SDOH processes—many of which are unjust—do not pertain to a given individual or group in isolation but are inherently relational in nature. They include policies, norms, and unjust social processes, such as racism, sexism, classism, xenophobia, and homophobia.^5^

We posit a reciprocal relationship between SDOH processes and SDOH capital. For example, it is well documented that structural racism contributes to persistent socioeconomic inequality experienced by communities of color, which includes differences in access to quality housing.^94^ At the same time, persistent socioeconomic inequality in communities of color in the United States has reinforced racist beliefs and attitudes that perpetuate structural racism.^95^ Evidence substantiates the impact of both social processes such as structural racism and a wide range of socioeconomic factors in shaping health inequities.^14^, ^48^, ^91^ Given that SDOH capital and SDOH processes shape health inequities together but also independently, they offer different opportunities for mitigation if conceptualized separately.

### Mediating Factors

We expand on the conceptualization of vulnerability as a primary proximal mediator of SDOH impact. Our organizing framework of SDOH mechanisms conceptually substitutes vulnerability with (1) environmental and behavioral *exposure* and (2) biological *susceptibility* (Box 1). The framework recognizes that health inequities emerge in communities in which either adverse exposure or susceptibility is elevated or in which both are elevated (the presence of proximal risk factors or the absence of proximal protective factors can increase adverse exposure). The work of Krieger as well as Chae and colleagues has contributed to this operationalization of exposure and susceptibility as substitutes for vulnerability.^26^, ^27^, ^39^ Notably, within this conceptual model, examining exposures without considering susceptibility and examining susceptibility without considering exposure each incompletely explains the emergence of population‐level health inequities, and application of the framework relies on the operationalization of both constructs.

### Moderating Factors

The proposed framework includes *resilience* as a moderator of the presumed mechanisms of SDOH. We conceptualize resilience as collective action that supports the ability of communities to thrive when confronted with structural challenges. Importantly, resilience represents a multilevel construct that does not solely rely on the strengths of individuals and communities and requires societal investments in the multilevel systems that are responsible for advancing health equity.^26^, ^27^, ^39^ SDOH mitigation efforts must not be limited to interventions designed to foster individual‐ or community‐level resilience. Application of the framework to explore any given health inequity therefore warrants operationalization of the micro‐, meso‐, and macrolevel resilience factors.

### Outcomes

Within the proposed framework, a given health inequity does not exist in isolation. In contrast to traditional, reductionist approaches, we conceptualize the impact of SDOH mechanisms on health inequities as dependent on the broader patterns of morbidity within the community of interest. As such, the framework calls for considering community and population health wholistically, taking cooccurring health inequities for which synergistic effects are hypothesized or empirically supported into account.

As a whole, the proposed heuristic framework is designed for application to a broad range of different health inequities. Therefore, the model accommodates broad classes of SDOH‐related causal factors, which can be operationalized more narrowly when the framework is applied to a specific health inequity of interest. Application of the framework includes the use of theory, empirical data, and the published literature to operationalize each depicted construct and evaluate the relevance of each depicted relationship (represented by arrows) in relation to a specified health inequity, place, and time.^96^ For example, the specific SDOH‐related causal mechanisms most relevant for explaining COVID‐19 inequities in an urban context in the United States may differ considerably from those most relevant for explaining stroke inequities in rural Australia. Nevertheless, in both scenarios, the proposed heuristic framework offers an aid to conceptualizing and operationalizing the key constructs and relationships to be considered. To illustrate, we discuss an exemplary application of the framework.

### An Example Application: Mitigating the Impact of COVID‐19 in the Bronx

The utility of the proposed framework as a heuristic for conceptualizing and operationalizing SDOH mechanisms becomes most apparent when an application to a specific health inequity is considered. To illustrate, we discuss an application of the framework to COVID‐19 mortality inequities in New York City's South Bronx. This example highlights a subset of well‐established SDOH influences and mechanisms that are important for understanding the selected health inequity. Additional factors undoubtedly contribute to COVID‐19 mortality inequities in the South Bronx but are omitted in our discussion because they would distract from the intended purpose of the example application, namely to illustrate the practical utility of our framework for conceptualizing and identifying specific SDOH mechanisms that can be targeted for intervention.

**Figure 3:**
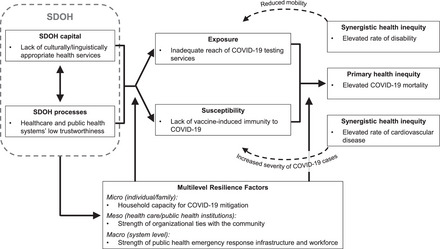
Exemplar Application of the Framework for Operationalizing SDOH Mechanisms that Shape COVID‐19 Mortality Inequities in the South Bronx, New York City. We illustrate how the framework introduced in Figure 2 can be applied to a specific health inequity. Here, we show example operationalizations for each construct depicted in Figure 3 for an application to COVID‐19 mortality inequities in the South Bronx, New York City. In addition, we show the conceptualized causal relationships (arrows). Notably, a causal effect of exposure on susceptibility, as included in Figure 2, is not supported by theory or data for the current example and is therefore omitted, but inclusion of the relational form should be considered (based on theory, empirical data, and the published literature) when the framework is applied to a different health inequity. We emphasize that the family context should be recognized as important for shaping the depicted constructs and relational forms (e.g., past experiences with health services among family members are associated with an individual's trust in health care providers^140^; health system literacy among parents can shape the receipt of health services for their children).^141^ The possibility of operationalizing framework constructs at multiple levels is illustrated here for resilience but can also be adopted for other constructs. Life‐course implications for COVID‐19 are not yet fully understood and are therefore not applicable for the current example, but the importance of a life‐course perspective for understanding the impact of SDOH on other health inequities is well established.^29^ Abbreviations: macro, macro level; meso, meso level; micro, micro level; SDOH, social determinants of health.

Since the beginning of the pandemic, more residents of Bronx County have died from COVID‐19 than in 99% of other counties in the United States,^97^ and the rate of COVID‐19 deaths in the Bronx is nearly twice the national average.^98^, ^99^ The South Bronx lies in the poorest congressional district in the continental United States and is designated as a medically underserved area with a shortage of health professionals.^100^, ^101^ The meaningful contribution of SDOH to elevated COVID‐19–related morbidity and mortality in similar socioeconomically and medically underserved contexts has been documented.^102^ In Figure 3, we apply our framework to illustrate operationalized mechanisms of influence for a well‐established *SDOH health capital* factor, namely a lack of culturally and linguistically appropriate health services,^103^ and well‐established *SDOH processes*, namely the health care and public health systems’ low trustworthiness among South Bronx residents.^104^

In the South Bronx, 97% of all residents are Latino or Black, and 30% are foreign born.^105^ Notably, the scarce health services in the medically underserved community are often inadequately aligned with the needs of its culturally and linguistically diverse residents, representing a major barrier to accessing health services and information^103^ (*SDOH of health capital*). At the same time, experiences with long‐standing, systemic barriers and unresponsiveness have rendered the extant systems for delivery of health care and public health services untrustworthy for communities experiencing structural racism and socioeconomic disadvantage^104^ (*SDOH health process*). Together, the two distal SDOH influences shape access to and meaningful engagement with health services among residents of the South Bronx.

In our example, the impact of SDOH on community‐level COVID‐19 mortality inequities is mediated by (1) reduced *exposure* to an important protective factor (i.e., inadequate reach of COVID‐19 testing services) and (2) increased biological *susceptibility* (i.e., lack of vaccine‐induced immunity to COVID‐19), each manifesting as a function of both less access to services and greater hesitancy to use available services.^106^, ^107^ The exposure (inadequate testing) influences the outcome (COVID‐19 mortality) through delayed COVID‐19 diagnosis and advanced disease progression before receipt of medical care.^108^ Lack of vaccine‐induced immunity represents increased susceptibility to severe COVID‐19 and risk of death. In this example, we consider the emergence of the primary health inequity of interest within the broader context of cooccurring health inequities in the South Bronx. Cooccurring, *synergistic health inequities*, such as elevated rates of disability, which reduces mobility and presents a barrier to accessing testing services,^109^, ^110^ and prevalent cardiovascular disease, which is associated with increased COVID‐19 severity,^111^ further exacerbate exposure and susceptibility.

Last, the example application operationalizes *resilience factors* at the individual/family, institutional, and system levels, which moderate the impact of SDOH on COVID‐19 mortality. For instance, household capacity for COVID‐19 mitigation, such as knowledge regarding the importance of COVID‐19 testing and vaccination and proficiency in navigating culturally misaligned health services, can moderate the effect of the distal SDOH factors on exposure and susceptibility. Operationalization of multilevel resilience factors is particularly important, given that they can significantly expand the number of leverage points for intervention that intuitively emerge from the application of the framework (e.g., in our example, family‐based interventions, institutional community engagement, and increased investments in public health infrastructure and the workforce).

We wish to highlight that the design and implementation of targeted SDOH mitigation programs based on the example discussed here is not only theoretically possible but that a real‐world application is currently in the field. The Nurse–Community–Family Partnership (NCFP) program, which is currently being evaluated in a community‐based, randomized controlled trial in the South Bronx as part of the National Institutes of Health (NIH)’s Rapid Acceleration of Diagnostics in Underserved Populations initiative,^112^, ^113^ is a family‐based intervention designed to increase COVID‐19 testing and vaccine uptake using a multilevel approach. In Table 2, we highlight several key NCFP program elements that target intervention opportunities for SDOH mitigation emerging from the example application of the framework in Figure 3. In addition, annotated photographs that provide impressions from NCFP implementation in the field are available online in the Supplementary Materials to this article.

**Table 2:** NCFP Program Elements Emerging from Applying the Heuristic Framework of SDOH Mechanisms in Figure 3

**Intervention Opportunity for SDOH Mitigation**	**Corresponding NCFP Program Element** **^a^**	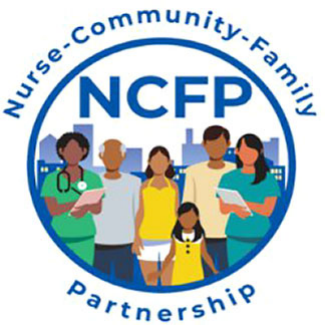
SDOH capital		
Lack of linguistically/culturally appropriate health services	Regular home visits by bilingual (English/Spanish) and bicultural nurse‐community health worker (CHW) intervention teams familiar with the community context; bilingual and culturally/contextually tailored printed NCFP intervention materials; bilingual nurse hotline	
SDOH processes		
Health care and public health systems’ low trustworthiness	Development of the NCFP Trustworthiness Agreement: a formal commitment of NCFP intervention teams to adhere to expectations in four dimensions of trustworthiness (transparency, respect, reliability, and benefit) that are collaboratively established with participating families at the beginning of the program	
Exposure		
Inadequate reach of COVID‐19 testing services	Offer of at‐home COVID‐19 testing to participating families for: Routine testing: monthly offer of at‐home polymerase chain reaction (PCR) test Indicated testing: same‐day at‐home rapid antigen test offered if COVID‐19 symptoms or exposure are reported	
Susceptibility		
Lack of vaccine‐induced immunity to COVID‐19	Theory‐based and culturally, linguistically, and contextually tailored intervention content designed to shape vaccine decision making; navigation to at‐home or community‐based COVID‐19 vaccination services	
Resilience factors		
Household capacity for COVID‐19 mitigation	Technical, knowledge, and skill components delivered by nurses (e.g., offer of COVID‐19 testing, training on household infection control skills, safe and correct use of PPE, etc.); theory‐based behavioral intervention components delivered by CHWs that address decision‐making factors (e.g., expectancies, norms, emotions, etc.)	
Strength of organizational ties with the community	Outreach to and engagement of key community partners to obtain buy‐in and establish a predictable relationship with the community (e.g., Tenants Associations)	
Strength of public health emergency response infrastructure and workforce	Development of sustainable COVID‐19 mitigation capacity through engagement and inclusion of community members in the NCFP implementation team and NCFP project team across all levels	
Synergistic health inequities		
Elevated rates of disability and cardiovascular disease	Screening for comorbidities, behavioral health, and social service needs among all family members; navigation to services when needed	

Abbreviations: NCFP, The Nurse–Community–Family Partnership; PPE, personal protective equipment; SDOH, social determinants of health.

^a^ NCFP is a community‐based, nurse and CHW‐delivered program designed to increase COVID‐19 testing, vaccine uptake, and overall household capacity for COVID‐19 mitigation. We highlight how several NCFP program elements directly address intervention opportunities for SDOH mitigation that emerge from the application of the heuristic framework of SDOH mechanisms in Figure 3. The NCFP evaluation study received funding from the National Institutes of Health (3P30 DA011041‐25S), ClinicalTrials.gov identifier NCT04832919.

## Conclusion

Despite increasing recognition of SDOH as the underlying drivers of historic and contemporary health inequities, a 2021 evidence review commissioned by the US Department of Health and Human Services found that the available set of rigorously evaluated and effective interventions to mitigate the impact of SDOH remains inadequate.^20^ The importance of a strong conceptual understanding of the precise mechanisms of SDOH influence for developing targeted and effective mitigation strategies has been argued by the World Health Organization and others.^31^, ^44^, ^114^, ^115^ Nonetheless, the SDOH frameworks that are most frequently applied in health care and public health planning and programming tend to rely on broad SDOH domains that are operationalized as being associated with a wide range of health inequities without adequate attention to the operating mechanisms. We propose an organizing framework that synthesizes and integrates eight unifying principles relating to the mechanisms of SDOH.

Our synthesis of the extant SDOH research is designed as a heuristic and customizable template for conceptualizing and operationalizing the specific SDOH mechanisms that represent potential leverage points for mitigating a given health inequity and serves as a tool to advance the translation of scholarly SDOH work into effective policy and programming (a continuously updated online tool to guide application of the framework is available at www.DUSONtrailblazer.com). As such, the framework aligns with calls for greater attention to the operating mechanisms shaping health inequities by NIMHD and other NIH institutes and offices.^116^, ^117^, ^118^ Further research should apply, empirically evaluate, and conceptually iterate on the framework of SDOH mechanisms proposed here, including by drawing on systems science perspectives that emphasize the dynamic processes shaping health inequities^119^ and through integration with other comprehensive, multilevel, but more static SDOH frameworks (e.g., the widely used NIMHD research framework).^32^ Moreover, work to develop, implement, and scale up programmatic applications for comprehensive SDOH mitigation is warranted, today more than ever. Over the past two decades, social inequality and health inequity have widened in the United States and in many other parts of the world.^120^, ^121^, ^122^, ^123^ Most recently, the ongoing COVID‐19 pandemic has disproportionately impacted communities experiencing preexisting social and health inequities worldwide.^102^, ^124^ These data suggest that new approaches to mitigate the impact of SDOH are needed. Frameworks that help target the mechanisms of SDOH more precisely and effectively will be indispensable if the increasingly broad and interdisciplinary initiatives designed to reverse the signs of deteriorating health equity are to be successful.

1

*Funding/Support*: This work was supported by the William T. Grant Foundation, Reducing Inequality Initiative, Grant 189030 (V.G.‐R.).

*Conflict of Interest Disclosures* V.G.‐R. reports grants and personal fees from ViiV Healthcare outside the submitted work, and he serves as a member of the US Presidential Advisory Council on HIV/AIDS and as a member of the CDC/HRSA Advisory Committee on HIV, Viral Hepatitis and STD Prevention and Treatment. The other authors declare they have no competing interests.

## Supporting information

SUPPORTING INFORMATION
