# Supplementary material for: Conceptualizing the Mechanisms of Social Determinants of Health: A Heuristic Framework to Inform Future Directions for Mitigation
Source: Milbank Q. 2023 Apr 16;101(2):486–526. doi: 10.1111/1468-0009.12642 (PMC10262397; doi:10.1111/1468-0009.12642)
Supplement: Supplementary file 1 — SUPPORTING INFORMATION [file MILQ-101-486-s001.pdf]

Supplementary Materials for

Conceptualizing the Mechanisms of Social Determinants of Health: A Heuristic Framework to Inform Future Directions for Mitigation

This PDF file includes:

Photos S1 to S5

Photo S1.

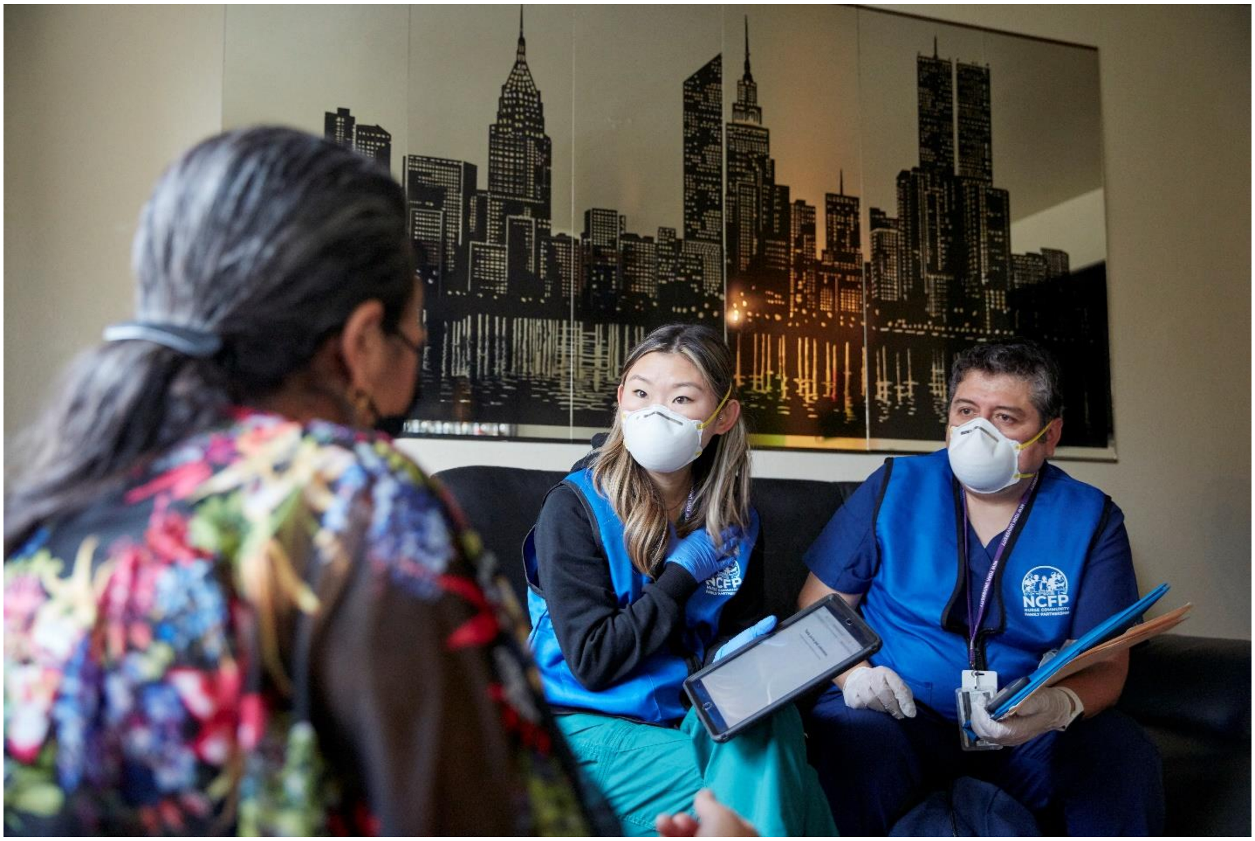

Photo Credit: Matthew Septimus

NCFP nurse (right) and community health worker (middle) review and discuss NCFP intervention materials with a family member during a home visit.

Photo S2.

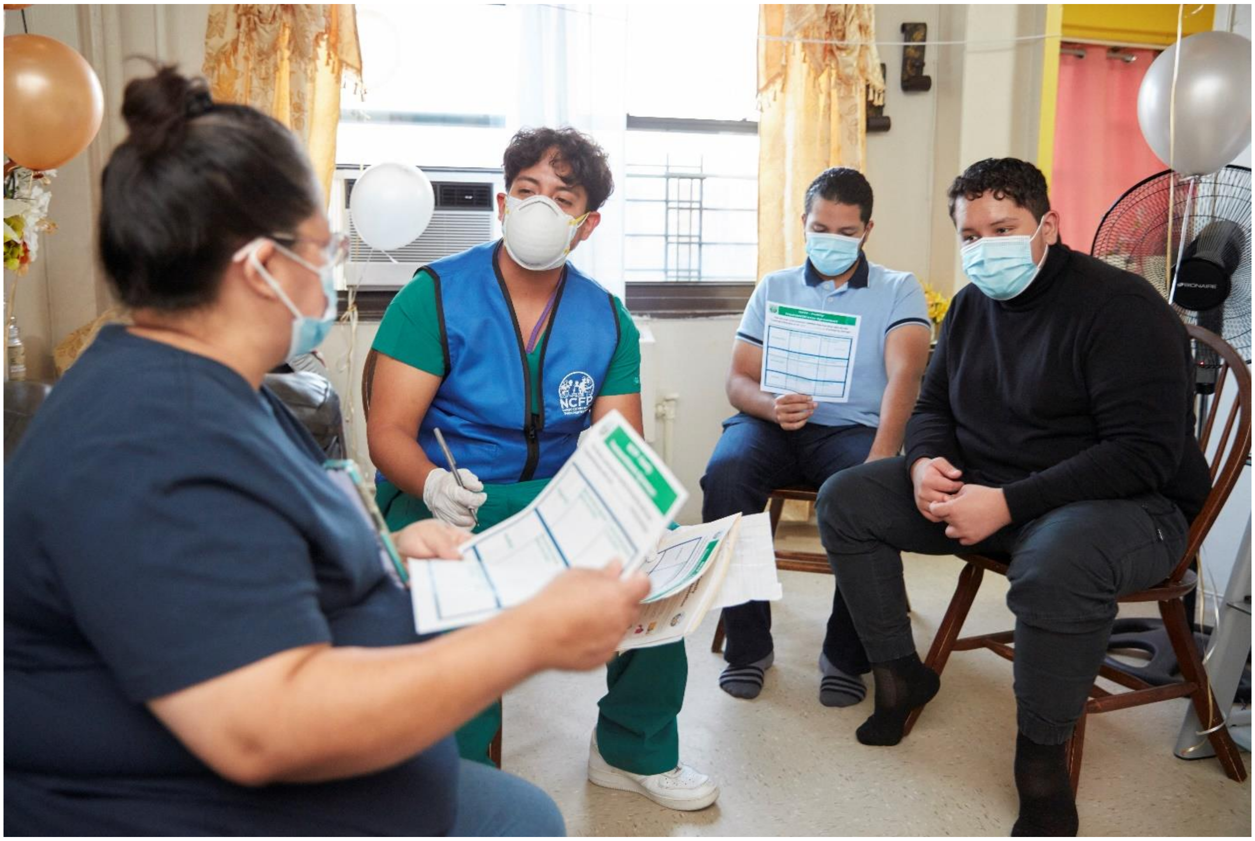

Photo Credit: Matthew Septimus

NCFP community health worker and a participating family collaboratively develop the NCFP Trustworthiness Agreement.

Photo S3.

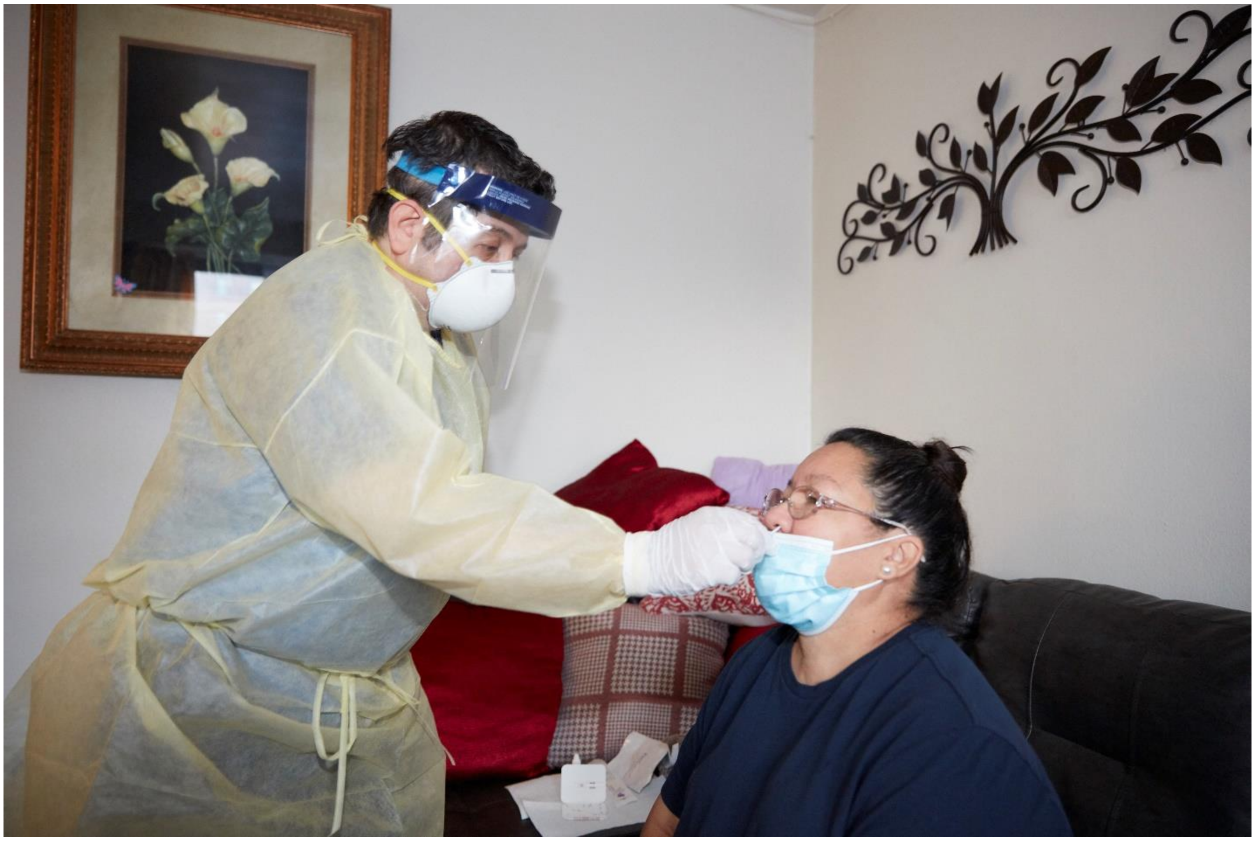

Photo Credit: Matthew Septimus

NCFP nurse collects a lower nasal swab for at-home rapid antigen COVID-19 testing (indicated testing offered if a participant experiences symptoms consistent with COVID-19 or an exposure).

Photo S4.

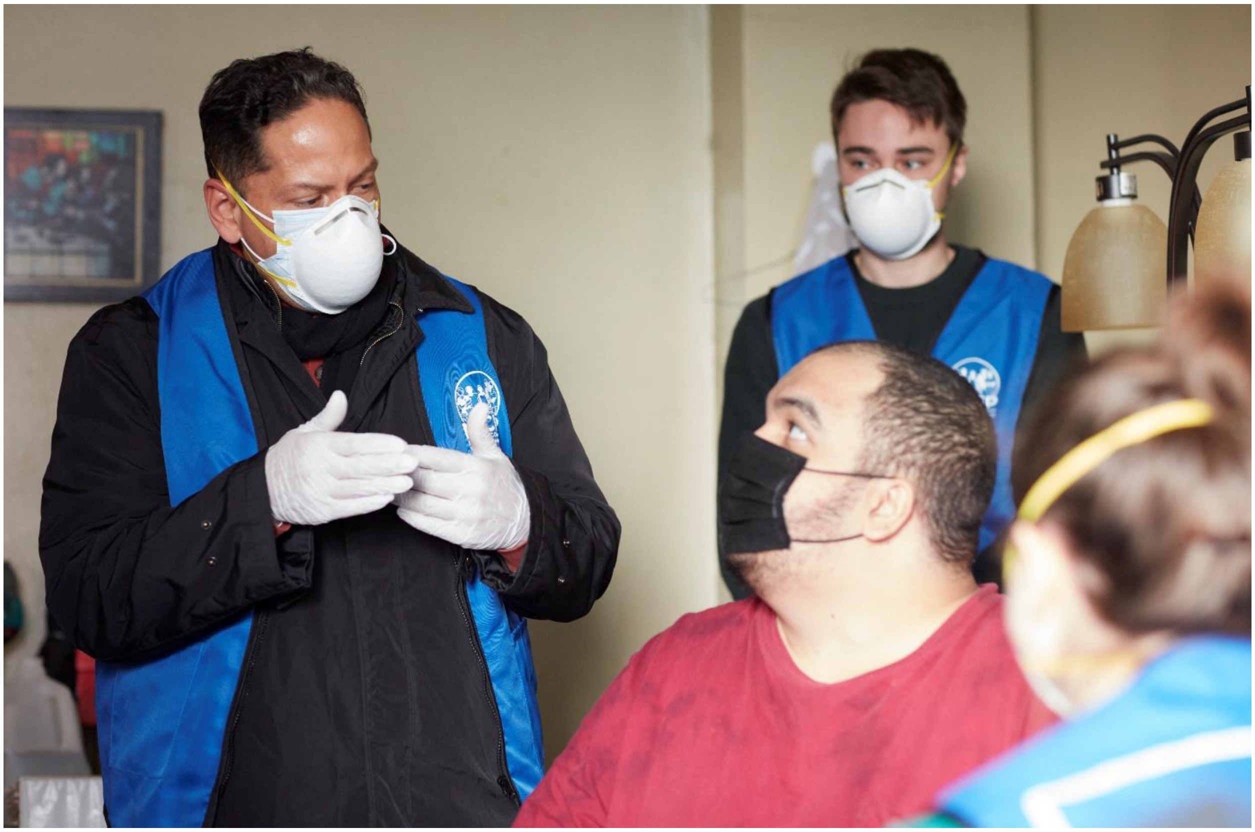
Photo Credit: Matthew Septimus

NCFP nurse provides guidance on household COVID-19 mitigation strategies.

Photo S5.

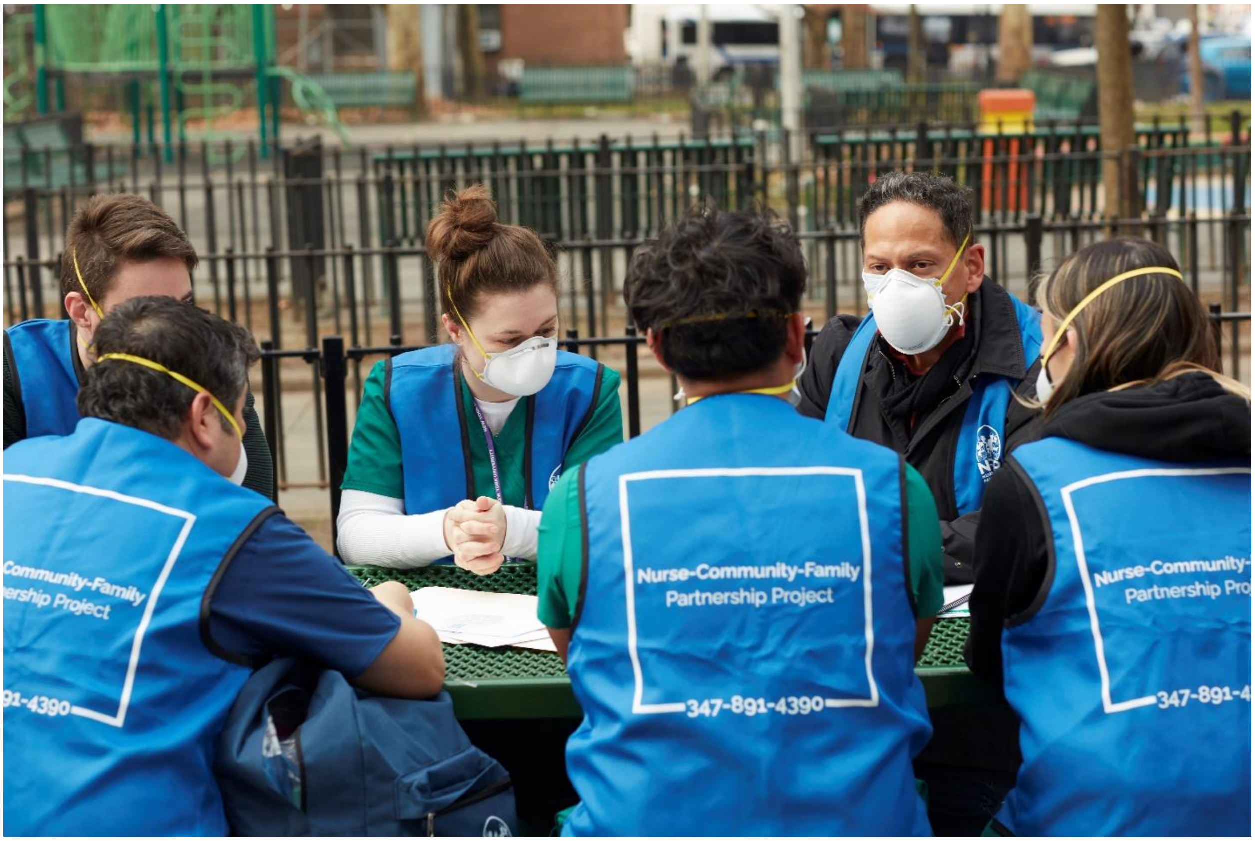

Photo Credit: Matthew Septimus

Team of NCFP nurses and community health workers meet in the South Bronx to coordinate family home visits.
